# The Use of Digital Health Interventions for Cardiometabolic Diseases Among South Asian and Black Minority Ethnic Groups: Realist Review

**DOI:** 10.2196/40630

**Published:** 2023-01-06

**Authors:** Aumeya Goswami, Lydia Poole, Zareen Thorlu-Bangura, Nushrat Khan, Wasim Hanif, Kamlesh Khunti, Paramjit Gill, Madiha Sajid, Ann Blandford, Fiona Stevenson, Amitava Banerjee, Mel Ramasawmy

**Affiliations:** 1 Institute of Health Informatics University College London London United Kingdom; 2 School of Psychology, University of Surrey Guildford United Kingdom; 3 Institute of Translational Medicine, University Hospital Birmingham Edgbaston United Kingdom; 4 Diabetes Research Centre, Leicester General Hospital, University of Leicester Leicester United Kingdom; 5 Warwick Medical School, University of Warwick Coventry United Kingdom; 6 Patient and Public Involvement Representative DISC Study United Kingdom; 7 University College London Interaction Centre University College London London United Kingdom; 8 Department of Primary Care and Population Health University College London London United Kingdom

**Keywords:** digital health, health inequality, cardiometabolic disease, cardiometabolic, ethnic, minority, cardiology, heart, eHealth, review, realist, context-mechanism-outcome, South Asia, Black, migrant, systematic search, literature search, African American, CVD, cardiovascular, diabetes, diabetic, type 2, mobile phone

## Abstract

**Background:**

Digital health interventions (DHIs) for the prevention and management of cardiometabolic diseases have become increasingly common. However, there is limited evidence for the suitability of these approaches in minority ethnic populations, who are at an increased risk of these diseases.

**Objective:**

This study aimed to investigate the use of DHIs for cardiovascular disease and type 2 diabetes among minority ethnic populations in countries with a majority of White, English-speaking populations, focusing on people who identified as South Asian, Black, or African American.

**Methods:**

A realist methodology framework was followed. A literature search was conducted to develop context-mechanism-outcome configurations, including the contexts in which DHIs work for the target minority ethnic groups, mechanisms that these contexts trigger, and resulting health outcomes. After systematic searches, a qualitative analysis of the included studies was conducted using deductive and inductive coding.

**Results:**

A total of 15 studies on the uptake of DHIs for cardiovascular disease or diabetes were identified, of which 13 (87%) focused on people with an African-American background. The review found evidence supporting the use of DHIs in minority ethnic populations when specific factors are considered in implementation and design, including patients’ beliefs, health needs, education and literacy levels, material circumstances, culture, social networks, and wider community and the supporting health care systems.

**Conclusions:**

Our context-mechanism-outcome configurations provide a useful guide for the future development of DHIs targeted at South Asian and Black minority ethnic populations, with specific recommendations for improving cultural competency and promoting accessibility and inclusivity of design.

## Introduction

### Background

Cardiovascular disease (CVD) and type 2 diabetes (T2DM; termed “cardiometabolic disease”) are common globally [1]. In the United Kingdom, CVD affects approximately 7 million people, is responsible for 1 in 4 premature deaths [2], and was the leading cause of death among males in 2018 [3]. T2DM is also highly prevalent, and the United Kingdom estimates suggest that the total number of adults with diabetes will surpass 4.6 million (9.5% of the population) by 2030 [4]. Therefore, cardiometabolic risk reduction is a key target of both primary and secondary prevention programs. Digital health interventions (DHIs) can support such programs by facilitating the diagnosis, management, and prevention of cardiometabolic diseases (CMDs) and improvement of overall health [5], as highlighted in the United Kingdom’s National Health Service Long-Term Plan [6]. Such health technologies are frequently grouped into 2 categories: eHealth, health services or information provided with the support of technology such as the internet, and mobile health (mHealth), including mobile phone apps, wearable devices, and SMS text messaging [7]. DHIs have been shown to reduce cardiometabolic risk factors and outcomes [8], improve glycemic control [9], and contribute to reduced hospitalizations as part of cardiac rehabilitation [10]; however, questions regarding their long-term clinical effectiveness remain.

In the United Kingdom, the 2 largest minority ethnic groups, those with a South Asian background and those with a Black background [11], are at an increased risk of CVD and T2DM compared with the rest of the population [12,13]; both groups have lower BMI cutoffs for diabetes risk than the White population [14]. Disparities in disease onset and outcome may be more prominent among the South Asian and Black minority ethnic groups in the United Kingdom, who may be more at risk of social and economic inequalities [15]. The increased risk of CMDs and of poorer outcomes among Black and South Asian minority ethnic groups has also been highlighted in North America [16-18]. Despite the high prevalence of CMDs and the number of digital tools available for prevention and management, evidence regarding the adoption and use of DHIs, specifically among South Asian and Black communities, is limited [19,20]. A lack of diversity in the planning and development phases of these technologies has been well documented [21,22]. In addition, lower health literacy [23], coupled with inequalities in digital access [24], may exacerbate the “digital divide” [25].

Frameworks to evaluate the uptake and implementation of DHIs exist, including models such as the Digital Health Engagement Model [26] and Nonadoption, Abandonment, and Challenges to the Scale-Up, Spread, and Sustainability [27]; some frameworks have been tailored specifically toward recognizing the needs of minority ethnic populations (eg, eHealth Equity Framework) [28]. However, questions around the use of DHIs by minority ethnic groups remain. Brewer and colleagues [29] set out a series of case studies in which DHIs were implemented in the United States using community-engaged research approaches to address concerns surrounding health inequity in relation to digital health care among minority ethnic groups. However, there is a lack of specificity in previous studies on DHIs that focus particularly on cardiometabolic health. In addition, more clarity is needed around the differences between and within minority ethnic groups to examine the nuances surrounding successful implementation and uptake. Owing to there being only a small number a published studies examining the use of DHIs among diverse ethnic populations in the United Kingdom, there is a need for studies that draw on cross-cultural comparisons, where recognition is given to the different political, social, and economic contexts likely to affect the patient experience and, subsequently, the generalizations that can be made to the United Kingdom setting. Therefore, we conducted a realist review [30] describing the use of DHIs for CMD, focusing on South Asian and Black minority ethnic communities in countries with a majority of White, English-speaking populations and where ethnic inequalities in health are well described, for example, the United Kingdom [31], the United States [32], and Canada [33].

### Objectives

Regarding the uptake and use of DHIs for CVD and T2DM among South Asian and Black minority ethnic groups, the objectives were to develop and refine the initial program theory (IPT) hypotheses based on the current literature, evaluate evidence, and develop a context-mechanism-outcome configuration (CMOc) to understand when, how, and why DHIs work.

## Methods

### Study Design

A realist framework was used [34], following RAMESES (Realist and Meta-Narrative Evidence Syntheses: Evolving Standards) guidelines [35], providing a rationale to explore complex differences in the uptake and use of DHIs among different ethnic groups by focusing on “what works for whom, in what circumstances, in what respects, and how?” [36].

### Development of IPTs

IPTs, which hypothesize how, why, and for whom the intervention may work [37], were compiled following a review of the literature and a parallel scoping review of frameworks that model the factors associated with the implementation and uptake of DHIs [38]. These IPTs considered the theoretical constructs related to the implementation, uptake, and use of DHIs (Multimedia Appendix 1 [27,28,39-46]) and were further refined into theory-driven hypotheses on the use of DHIs among minority ethnic groups, where “use” was defined to capture acceptability, relevance, and user experience (Textbox 1).

**Refined initial program theories (IPTs) regarding the use of digital health interventions (DHIs) among ethnic minority groups.**

- Individual factors—factors centered on the end user
  IPT-1: Beliefs and personal views or perceptions (whether accurate or not) held around the use of DHIs in particular and technology in general will shape an individual’s use of DHIs.
  IPT-2: An individual’s education and literacy levels will affect their ability to use DHIs.
  IPT-3: The prevailing physical health conditions and associated functional disability will shape an individual’s use of DHIs.
  IPT-4: An individual’s beliefs are, at least in part, determined by their social networks and the beliefs of significant others; therefore, the use of DHIs is shaped by social experiences and interactions.
  IPT-5: An individual’s material circumstances shape their use of DHIs.
- Technology factors—factors related to the technological aspects of DHIs
  IPT-6: The functional and performance-related aspects of DHIs will shape an individual’s personal use of DHIs.
- Community factors—factors related to the community in which the individual resides
  IPT-7: The community in which an individual lives can support the use of DHIs, if appropriately resourced.
- System factors—factors related to the wider governing system
  IPT-8: The national and regional health care system that an individual is part of will influence (in some circumstances) the use of DHIs.

### Search Strategy and Selection Criteria

#### Overview

Studies that evaluated the acceptability or use of DHIs aimed at improving or managing the cardiometabolic health of individuals from a South Asian or Black minority ethnic group living in countries with a majority of White, English-speaking populations were included. All study designs were included, including cross-sectional studies and those using both qualitative and quantitative methods. Inclusion was limited to studies published in English and those carried out 10 years before the search to obtain the most contemporary evidence. Multimedia Appendix 2 provides the full inclusion and exclusion criteria.

#### Information Sources

Literature searches were conducted using the web-based databases PubMed, Scopus, and Google Scholar in June 2021, and they were repeated in December 2021 (Multimedia Appendix 3). Because both ethnicity and CMD have relevance in a range of contexts, gray literature was additionally obtained through Google Scholar and open-access resources, including “medRxiv,” “arXiv,” conference proceedings, and reference lists by AG.

#### Study Selection

**Figure 1:**
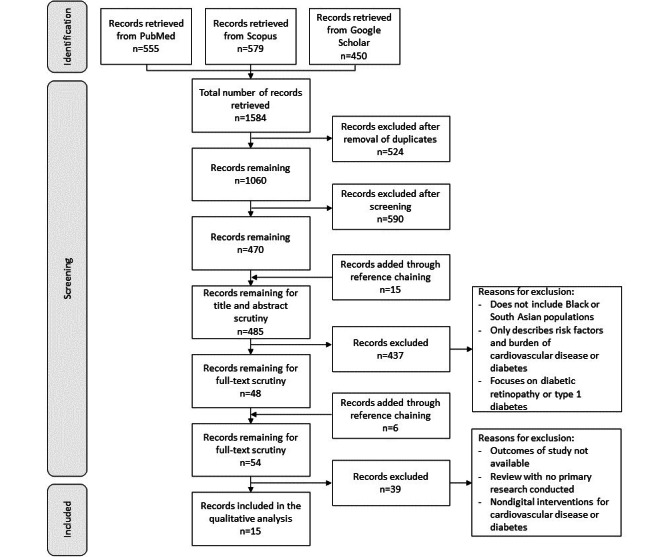
PRISMA (Preferred Reporting Items for Systematic Reviews and Meta-Analyses) diagram illustrating the process of searching for articles to be included in the review.

Deduplicated search results were imported into Zotero [47], and studies were selected after title and abstract screening, which was followed by full-text screening, against the inclusion and exclusion criteria (Figure 1). The screening was performed by AG, with feedback from and discussion with LP and MR. Any disagreements were resolved by discussion to reach a consensus, and reasons for exclusion were recorded.

### Data Analysis

Data charting of the final studies included details regarding the citation, methods (sample size, participant [or patient] group, and their health issues with regard to CVD or T2DM), recruitment criteria, purpose of the DHI, context (ie, country and place of data collection), and key findings, including relevant statistics where applicable.

The included studies were imported into NVivo (version 12, QSR International) for qualitative analysis [48]. An iterative process of both inductive and deductive (informed by our IPTs) analyses was carried out and used to create CMOcs, which describe how “contextual factors (C) work to trigger particular mechanisms (M), and how this combination generates various outcomes (O)” [49], in this case, how cardiometabolic DHIs work, or are expected to work, within specific minority ethnic groups, identifying conditions that may hinder or promote successful outcomes [50].

## Results

### Overview

The PRISMA (Preferred Reporting Items for Systematic Reviews and Meta-Analyses) diagram in Figure 1 displays the search and selection process for inclusion, which culminated in a total of 15 included articles.

The key characteristics of these studies are presented in Table 1. The included studies had one of two aims: either to explore the views on and experiences of using DHIs for CVD and T2DM (7/15, 47%) or to establish the health benefits of engagement with such DHIs (8/15, 53%). Most (13/15, 87%) study methods required participants to engage with a DHI as part of either an intervention or interactive testing and feedback. The sample size reflected the range of research methods used across the studies, including qualitative interviews (n=9) and clinical trials (n=343; mean 58, SD 84; median 30). Overall, 60% (9/15) of studies focused on CVD, whereas 40% (6/15) of studies focused on T2DM. All the studies recruited participants from a minority ethnic background: South Asian background (2/15, 13%), one in Canada and the other in the United Kingdom, or African-American background (13/15, 87%). Most (11/15, 73%) of the included studies among African-American populations focused their recruitment on low-income areas with a high burden of CVD and diabetes; this information was not provided for the study focusing on the South Asian populations in Canada [51]. The study focusing on the South Asian populations in the United Kingdom sampled across population factors, including age, education, migratory generation, and occupation [52]. Of the 15 studies, 8 (53%) recruited participants through opportunistic and convenience sampling from places such as faith centers, health care settings, and community centers or those in specific communities, and the remaining 7 (47%) studies recruited participants from hospital or primary care records. Some studies were theory driven, using the health belief model, a community model such as community-based participatory research, the social cognitive theory, or social modeling [53-56].

**Table 1:** Summary of studies included in the review (N=15).

Title		Author, year	Sample size	Health focus	Recruitment criteria (as defined by the study authors)	DHI^a^ purpose	Country	Methods of data collection	Digital requirements needed to participate in the study	Principal findings
**Studies exploring the views on and experiences with DHIs**										
	Community Engagement in the Development of an mHealth-Enabled Physical Activity and Cardiovascular Health Intervention (Step It Up): Pilot Focus Group Study	Ceasar et al [57], 2019	16	CVD^b^	African-American individuals at risk of CVD, as they were recruited from an area with the highest burden of CVD in Washington, DC	mHealth^c^ app containing motivational messages and educational content to promote physical activity with the aim of reducing the risk of developing CVD	United States	Interviews and focus groups	Participants were required to own a smartphone and were provided with a Fitbit Charge 2 device	The main themes emerging from the focus groups and interviews include perceived benefits, perceived barriers, perceived susceptibility, cues to action, and self-efficacy
	Young African American Women’s Participation in an mHealth Study in Cardiovascular Risk Reduction: Feasibility, Benefits, and Barriers	Kathuria-Prakash et al [58], 2019	40	CVD	African-American females at risk of CVD, as they had at least two risk factors	mHealth intervention included 4 sessions of CVD risk reduction education and 6-month smartphone CVD risk reduction monitoring	United States	Self-complete questionnaires	Participants received a smartphone device and wireless blood pressure machine for the study	60% (n=23) of participants reported that the interventions were easy or very easy to maintain, and 90% (n=35) reported that the app was easy or very easy to use. A total of 60% (n=24) of the participants observed that their family’s nutrition improved “a lot” or “a medium amount,” and many participants noted positive changes in their children’s diets. Only 8% (n=3) of the participants cited the time or cost required to prepare healthy foods as barriers to implementing dietary changes
	SMARTWOMAN: Feasibility Assessment of a Smartphone App to Control Cardiovascular Risk Factors in Vulnerable Diabetic Women	Wenger et al [59], 2019	14	CVD	African-American individuals with known T2DM^d^ but no known CVD	The use of a Fitbit to control cardiovascular risk factors such as blood glucose levels, daily steps, and blood pressure as well as SMS text message reminders and encouragement for using the Fitbit	United States	Self-complete questionnaires	Smartphones and Fitbits were provided to all participants for the purpose of the study	The use of this app resulted in high patient satisfaction and motivation for participants, and it reinforced healthy behavior regarding cardiovascular health
	A Cardiovascular Health and Wellness Mobile Health Intervention Among Church-Going African Americans: Formative Evaluation of the FAITH! App	Brewer et al [53], 2020	9	CVD	African-American individuals with CVD risk factors due to their ethnicity	mHealth (app) to promote CVD health by tracking physical activity and diet and providing health information from cardiovascular health care professionals	United States	Focus groups	N/A^e^	FAITH! app users reported high satisfaction and a positive impact on health-promoting behavior, improving their cardiovascular health; cultural tailoring, education modules, and social network were facilitators of the success
	Time to Listen: a Mixed-Method Study Examining Community-Based Views of Mobile Technology for Interventions to Promote Physical Activity	Claudel et al [54], 2020	16	CVD	African-American females who had obesity or were overweight and thus regarded as at risk of CVD	This study investigates the use of mHealth DHIs through focus groups and surveys	United States	Focus groups	N/A	Participants had a high attachment to mobile phones and high technology adoption. The need for cultural and community-level customization and collaboration with the community was emphasized
	British South Asian Patients’ Perspectives on the Relevance and Acceptability of Mobile Health Text Messaging to Support Medication Adherence for Type 2 Diabetes: Qualitative Study	Prinjha et al [52], 2020	67	T2DM	South Asian individuals with prediagnosed T2DM	This study explored participants’ opinions on the use of mHealth (SMS text messages) through focus groups to aid the development of future SMS text message interventions for this population group	United Kingdom	Focus groups	N/A	Short messages are acceptable; messages should meet their cultural needs such as information about South Asian foods. Short messages delivered in English are acceptable because family members often translate, but different formats may be needed, for example, videos. Face-to-face interaction is needed for those who do not use digital devices
	Perspectives From Underserved African Americans and Their Health Care Providers on the Development of a Diabetes Self-management Smartphone App: Qualitative Exploratory Study	Barber-Gumbs et al [60], 2021	78	T2DM	African-American individuals who had prediabetes or T2DM. Their family, friends, and health care providers were also included in the study	This study explored how a smartphone app can be used to improve diabetes self-management in terms of desired features and potential improvements to mock-up apps	United States	Forums, focus groups, and interviews	Participants were smartphone users	Participants mentioned that apps could help people who cannot easily access health care, specifically to provide diabetes education and self-management. Tracking diabetes care–related behavior and receiving feedback on progress was also mentioned as a way to increase engagement with self-management
**Studies exploring the health benefit of DHIs**										
	Feasibility and Usability of a Text Message-Based Program for Diabetes Self-management in an Urban African-American Population	Dick et al [61], 2011	18	T2DM	African-American individuals diagnosed with T2DM	Personalized mHealth as an SMS text message to improve diabetes self-management	United States	Surveys and interviews	Participants must have owned a smartphone but received US $25 to cover expenses for an unlimited SMS text message plan	Participants increased their confidence in diabetes self-management, and missed medication doses decreased after the intervention
	Mobilizing Your Medications: An Automated Medication Reminder Application for Mobile Phones and Hypertension Medication Adherence in a High-Risk Urban Population	Patel et al [62], 2013	50	Prediagnosed hypertension and T2DM	Individuals with Medicaid as primary insurance, using at least 2 prescription medications for hypertension; most participants were diagnosed with T2DM, 96% were African-American	mHealth intervention as a mobile phone reminder for participants to take their medications; blood pressure and pharmacy refill records were assessed throughout the study	United States	Self-report questionnaires	Participants were given mobile phones preloaded with the “Pill Phone” app	Participants reported a high level of satisfaction with the intervention and increased medication adherence. Adherence, as measured by pharmacy recall data, showed a trend toward improvement, and it declined significantly after the intervention was discontinued
	Diabetes Island: Preliminary Impact of a Virtual World Self-care Educational Intervention for African Americans With Type 2 Diabetes	Ruggiero et al [63], 2014	41	T2DM	African-American individuals diagnosed with T2DM	An eHealth, virtual world intervention to improve diabetes self-care, clinical outcomes, and psychosocial factors and be seen as useful for patients with T2DM	United States	Self-report questionnaires	Participants were provided with a laptop that contained only the virtual program “Diabetes Island”	This intervention demonstrated participant acceptability; regular use; and promising outcomes, including improvements in BMI, diabetes-related distress, physical activity, dietary intake, and environmental barriers to self-care. The responses regarding the acceptability and usefulness of Diabetes Island were consistently positive
	A Digital Health Intervention to Lower Cardiovascular Risk: a Randomized Clinical Trial	Anand et al [51], 2016	343	CVD	South Asian individuals who were overweight or obese and had the ability to physically engage in study activities	eHealth to examine the MI^f^ risk score in those who may not currently have CVD but are at risk of developing CVD owing to their ethnicity	Canada	Analysis of MI risk score	Participants were required to own a smartphone device	DHI use was not associated with a reduction in MI risk score after 12 months and was not influenced by knowledge of genetic risk status
	Adherence with Physical Activity Monitoring Wearable Devices in a Community-Based Population: Observations From the Washington, D.C., Cardiovascular Health and Needs Assessment	Yingling et al [55], 2017	99	CVD	African-American individuals who were at risk of CVD, as they were from communities of Washington, DC, with high obesity rates and low household incomes	mHealth (wristband) to track CVD health measures	United States	Self-reported questionnaires	Participants were given a physical activity–monitoring wristband	mHealth systems with a wearable device and local data collection hub may feasibly target resource-limited communities. No significant difference in CVD health factors were found between users and nonusers of the intervention
	A Smartphone App for Self-management of Heart Failure in Older African Americans: Feasibility and Usability Study	Heiney et al [64], 2020	12	CVD and heart failure	African-American individuals diagnosed with heart failure	Baseline and postintervention comparisons were made based on 4-week use of a health app to promote the self-management of heart failure	United States	Interviews and statistical analyses	N/A	The app did not significantly increase the quality of life but did show clinically relevant changes in heart failure self-care maintenance, management, and confidence
	Development and Evaluation of a Tailored Mobile Health Intervention to Improve Medication Adherence in Black Patients with Uncontrolled Hypertension and Type 2 Diabetes: Pilot Randomized Feasibility Trial	Schoenthaler et al [65], 2020	10	T2DM	Black individuals who had uncontrolled hypertension and T2DM and at least one CVD risk factor	mHealth intervention that includes surveys, interactive modules, and educational modules to improve medication adherence to reduce hypertension and manage T2DM	United States	Interviews and self-report questionnaires	N/A	After a 3-month mHealth intervention, medication adherence improved, but there were no significant decreases in systolic blood pressure and HbA_1c_^g^ (average blood sugar levels). High acceptability of mHealth intervention
	Results of a Culturally Tailored Smartphone-Delivered Physical Activity Intervention Among Midlife African American Women: Feasibility Trial	Joseph et al [56], 2021	20	CVD	African-American females who had a BMI ≥30 kg/m^2^ and did ≤60 minutes of moderate-to vigorous-intensity physical activity per week	4-month physical activity intervention to increase physical activity including the use of an app, Fitbit, and SMS text messages	United States	Focus groups and tracking of Fitbit wear and progress	Participants were provided with a Fitbit Alta HR for the study	Participants increased moderate-to-vigorous physical activity per week. Of the 15 participants who completed the satisfaction survey, 14 (93%) indicated that they would recommend the intervention to a friend. Social support could improve the intervention

^a^DHI: digital health intervention.

^b^CVD: cardiovascular disease.

^c^mHealth: mobile health.

^d^T2DM: type 2 diabetes.

^e^N/A: not applicable.

^f^MI: myocardial infarction.

^g^HbA_1c_: hemoglobin A_1c_.

These studies reported both the behavioral and clinical effects of DHIs. Behavioral effects included an increase in physical activity assessed by energy expenditure according to the REGICOR model [56], a reduction in hospital readmissions coupled with improved heart failure self-care maintenance and confidence [64], and an increase in daily steps [59]. The clinical effects of DHIs included improvements in blood pressure [62,65] and a decrease in blood glucose levels or hemoglobin A_1c_ [59,63]. However, Schoenthaler et al [65] found no effect of their DHI on medication adherence among African-American ethnic groups, and the DHI evaluated by Anand et al [51] was unsuccessful in reducing the myocardial infarction risk score among South Asians.

### Evidence for IPTs

IPT 1 suggests that an individual’s beliefs and perceptions about the use of DHIs and technology shape their use of DHIs. The included studies did not directly explore participants’ beliefs before their enrollment in the study. However, their willingness to participate in studies investigating the use of DHIs suggests a predilection for technology. The perceived utility of DHIs was partly dependent on the participants’ ability to integrate the intervention into their daily lives; for example, a DHI to support medication adherence and make lifestyle changes showed high acceptability and benefits to participants by helping them develop habitual behaviors and overcome disruptions to daily routines [65]. IPT 1 also recognizes how individual perceptions shape the ongoing use and interaction with DHIs. Joseph et al [56] examined the change in self-efficacy from baseline to 4-month follow-up in midlife African-American women. Contrary to their hypothesis, they found that self-efficacy decreased over the course of the study, with one participant saying, “I wish I had been as active as I inspired[sic] to be.” This indicates that participants may reappraise the perceived benefits of DHIs over time relative to costs, such as time constraints and caring commitments.

The studies included in this review showed mixed support for IPT 2, which focuses on how users’ education, literacy, and digital skills might impact their engagement with and use of DHIs. For example, some studies recognized the impact of low digital literacy and skills on successful engagement with DHIs in participants from both African-American and South Asian backgrounds [52,54,61,62]. Both Claudel et al [54], 2020, and Dick et al [61], 2011, reported that app design and user interface are aspects that might act as potential barriers for users with low digital literacy. To support participants in interacting with “Diabetes Island,” which used an existing virtual world platform for diabetes education, the research team added in-world technical support as well as provided additional materials on the study website and via telephone [63]. However, a study of an mHealth physical activity–monitoring system in an African-American community found no differences in technology fluency between users and nonusers, suggesting that all participants had high digital literacy on study entry [55]. The participants also expressed a preference for the use of layman’s terms instead of medical jargon in the descriptions of health content. DHIs that were positively reviewed by participants of all ethnicities included those that provided education modules that were easy to understand, including DHIs for cardiovascular wellness [53], diabetes medication adherence [52], and increasing physical activity [54].

IPT 3 indicates that the prevailing health conditions shape an individual’s uptake and use of DHIs. DHIs included in this review targeted different patient groups. For example, 27% (4/15) of studies recruited participants from populations at high cardiovascular risk for physical activity interventions [54-56,66], and 20% (3/15) of studies recruited people with existing disease (hypertension or diabetes) to improve medication adherence [52,62,65]. The included studies described a complex picture of interactions between the existing disease risks or states and the motivation to engage with DHIs or behavior change. Some participants expressed positive engagement with the interventions: “I didn’t lose weight, but it showed in my blood tests...So, I did show some improvement with the increasing of the exercise.” [57]. A study using an SMS text message and email DHI to reduce cardiovascular risk in South Asians in Canada found that at 1-year follow-up, there was no evidence for behavior change because of the intervention and suggested that this might be because of the existing healthy behaviors of participants at study entry [51]. Conversely, a study of the use of a physical activity–monitoring system in a population at risk of CVD in the United States found that the participants who did not smoke and were physically active before the intervention were more likely to engage with a physical activity–monitoring system [55].

IPT 4 focuses on the interaction between individuals’ beliefs, social networks, and DHI use. Some authors have noted the importance of personal networks in enabling digital access. For example, South Asian participants in the study of Prinjha et al [52] stated that “older people don’t even have mobile phones” and highlighted the importance of family members acting as key stakeholders in these patients’ access to health care via DHIs. Other evidence in support of IPT 4 considers participant engagement with DHIs and its impact on participants’ social networks. Peer support during the intervention phase of the study was demonstrated to be beneficial. For example, Brewer et al [53] stated that “participants enjoyed sharing and learning from each other through a variety of communication modalities on the sharing board (e.g., text, pictures, videos).” However, the provision of solely a discussion board may not be sufficient; participants in a physical activity intervention expressed a desire for the research staff to increase their engagement with participants and organize group exercises [56]. Wider benefits of the engagement with DHIs were also observed in some studies. For example, Kathuria-Prakash et al [58] found that >60% of 38 participants reported that their family’s nutrition positively changed “a lot” or a “medium amount” because of their participation in the study.

IPT 5, which states that an individual’s material circumstances shape their use of DHIs, was supported in some instances. Anticipating this during the design of the study, 47% (7/15) of studies provided participants with devices [55,56,58,59,62-64], with 7% (1/15) explicitly stating that this was to minimize barriers to participants' access to the DHI [63]. The study results reported mixed findings concerning DHI use and the socioeconomic status of participants. In their study of the use of a hub-based mHealth physical activity–monitoring system in resource-limited communities, Yingling et al [55] reported that individuals with lower socioeconomic status were more likely to use the system over a 30-day period than those with a higher socioeconomic status. In a mixed methods study with African-American women, the benefits of mobile DHIs were listed as convenience, efficiency, and cost-effectiveness—a participant explained how she “saves a lot of gas money” by using her phone to undertake tasks she previously used to complete in person [54]. Regarding financial constraints affecting the engagement of participants with the content of DHIs, a preventive cardiovascular mHealth program in young African-American women found that only 8% (n=3) of the participants said that the cost of healthy foods was a barrier to their behavior change from the DHI [58]. Similarly, participants in a cardiovascular mHealth intervention among African Americans acknowledged that eating more healthily can sometimes be more expensive, but the value of eating healthier to improve overall health outweighed the extra costs [53].

IPT 6 describes the technical and design factors of DHIs that impact individuals’ willingness to initiate and maintain use; this was supported in our review in two ways. First, the studies identified technical factors that can improve the use of the DHI, namely the inclusion of a search function within the app and the option to integrate the DHI with other apps or devices [53,60,66]. It was recognized that using a DHI was convenient as participants were attached to their phones “at all times and locations” [54]. Of the 15 included studies, 4 (27%) reported technical difficulties affecting the user (eg, difficulties with accessing all parts of the app, cumbersome external material, and malfunctions), and some provided staff to support participants [53,57,62,64].

Second, the simplicity of the design of DHIs, such as the user interface, user experience, and visual aspects, proved to be important for their continued use across all study types [53]. When working with a digitally naive population with low literacy, Heiney et al [64] identified that careful consideration of the phone display (ie, simple and uncluttered) and the use of clear instructional materials were essential. Pictorial and visual information in the form of videos, screenshots, and pictures with minimal text was identified to be important for users to promote social modeling [52-54,56,64,66], particularly for participants with low literacy [52]. Similarly, reducing the amount of information provided via DHIs or frequency of notifications was favored by participants across study types [52,53,64,65]. One of the participants said, “If we’re going to get too many of these messages, people are just going to ignore them...” [52]. Dick et al [61] found that the tapering of information was useful, with more information provided to the user at the start followed by phasing out of information once a routine was established.

All the DHIs included in this review promoted behavior changes in the user. Such behavioral changes were incentivized via a variety of methods. Positive feedback encouraged users to persist with the self-management of both diabetes [60] and cardiovascular health [53]. Others used “prizes” in the form of healthy recipes [66]. These personalized aspects of DHIs were found to encourage continued use and allowed greater self-monitoring of one’s health [53,54,56,59,60,64-66]. However, behavior change was not always successful, even with an incentive [65].

Easily accessible health education was extremely important for users, including information regarding disease physiology [51,53,56,58,60,62,65] and affordable local resources [60], learning was enhanced by interactive components such as quizzes [53,65]. Elements that allowed users to track progress and set goals and reminders were identified as important and useful within DHIs [56,60] and increased participants’ sense of accountability to meet personal goals and remain active [53,54,56,60,61,64,66]. Interestingly, this level of interactivity was not provided by the DHI in the study conducted by Anand et al [51], which was unsuccessful in improving cardiac risk scores in South Asian groups.

The suitability of apps and cultural competency of their content were also considered in the included studies. Interviews with African-American participants with hypertension and T2DM identified tailored strategies for medication adherence, which included collaborative patient-provider communication, the use of peer vignettes, and stress reduction techniques [65]. The participants in 15% (2/13) of studies in African-American populations expressed preferences for content to be representative of their identities and characteristics to which they belong, such as race or ethnicity, body size, and physical capacity and the community-level tailoring of content and resource [53,54] In addition, 15% (2/13) of studies identified the importance of spirituality in the African-American culture of their participants and incorporated aspects such as biblical quotes or health promotion within the church congregation to increase the relevance of their intervention [53,64]. Similarly, South Asian participants suggested that DHIs should include culturally adapted messages about foods, herbal approaches, fasting, and exercise in women-only groups and the use of images and audio messages for patients unable to read message content in English rather than translated written content [52].

The integration of community-specific information greatly improved user response to DHIs, which lends support for IPT 7. In-app connections to the existing community resources were important to participants, such as location-specific recommendations for outdoor physical activity [66] and an interactive map of the local area and where to find health information [65]. Information from trusted sources such as health care professionals was identified as a strong motivator to continue using DHIs [52,53,61]. The presence of community support within DHIs to share stories and testimonials from people of the same ethnic group was shown to be beneficial to DHI use and increase self-efficacy [53,56,58,60,65,66], building on the findings from IPT4, about the role of social experiences shaping the use of DHIs. In a study that did not include a community facility, 1 participant mentioned that they would not recommend the intervention to a friend, as it “didn’t allow for building fitness connections,” underlining the need for social support in DHIs [56].

Regarding IPT 8, although the studies included in our review did not explicitly investigate the role of the health care system in the use of DHIs, there was some indication that the integration of the user’s health care professional and the option to share information from the DHI with their health care professional would serve to improve the intervention [52,53,61]. Participants in a cardiovascular wellness intervention reported that using the DHI improved their relationship with their health care provider, as they were more informed and prepared during regular checkups [53], and among participants with previously uncontrolled diabetes and hypertension, the ability to share results about medication adherence with their physician helped stimulate conversations about challenges to adherence on their next clinical visit [65].

**Figure 2:**
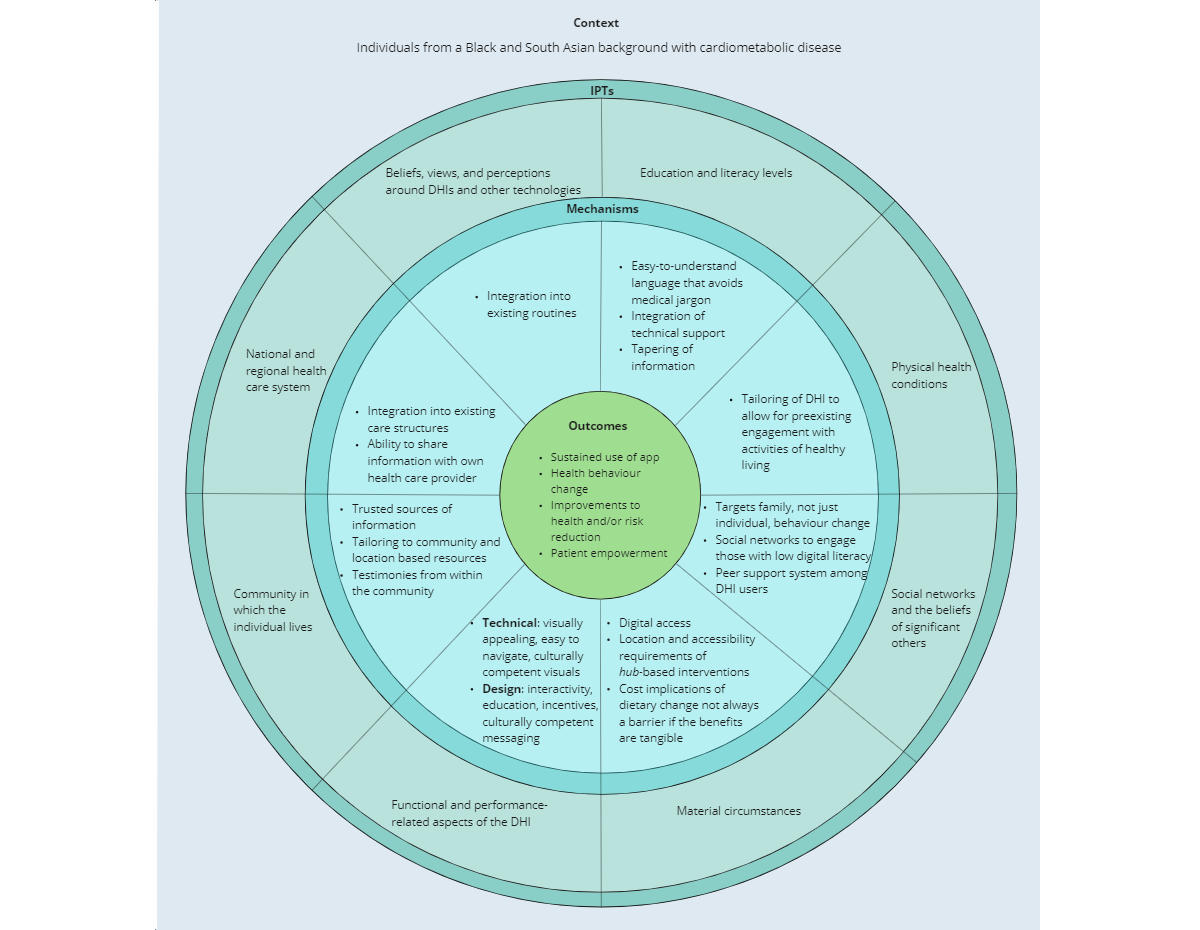
Context-mechanism-outcome configurations (CMOcs) for digital health interventions (DHIs) for cardiometabolic diseases among South Asian and Black ethnic minority groups. IPT: initial program theory.

The assimilation of evidence in relation to our 8 IPTs led us to formulate CMOcs, which we have represented in Figure 2. We broadly define the “context” within this configuration as individuals who self-identify as belonging to a South Asian or Black minority ethnic group in a country with a majority of White, English-speaking populations. Furthermore, according to Figure 2, we propose that each IPT directly maps onto corresponding “mechanisms,” which in combination contribute to the desired “outcomes” of DHIs. We postulate that the mechanisms through which our IPTs effect change in the outcomes include the integration of DHIs into the existing routines and lifestyles of patients, avoidance of jargon and medical language, targeting of behavior change to the wider family, recognition of peer support systems, promotion of accessibility in terms of both physical location and cost, technical and design aspects of the DHI interface and content, leveraging of community resources, and integration of DHIs into the existing care structures. In summary, the outcomes of DHIs included the sustained use of the interventions, health behavior changes, improvements to health or risk reduction, and patient empowerment; however, the exact outcomes vary according to the scope of the specific DHI.

## Discussion

### Findings in Context

In this first realist review of the use of DHIs for CMD among South Asian and Black minority ethnic groups, we found that a culturally competent design of digital health can promote the uptake, use, and benefit of digital health approaches in minority ethnic populations. We reviewed 15 primary research studies in relation to our 8 IPTs, which consider how individual, technology, community, and system factors affect the use of DHIs in minority ethnic groups. We largely found evidence in support of our IPTs; if evidence was found to be contradictory or inconsistent, it was because of the differences across study designs, including the inclusion and exclusion criteria applied to participant recruitment. We further applied the realist approach through the production of CMOcs in which we described the translation of each IPT into an outcome via different mechanisms of action.

DHIs for CMDs are wide ranging and include approaches to reduce disease risk through behavioral or lifestyle changes [8], as well as approaches specifically designed to help those with an established disease to manage their symptoms and mitigate future complications or disease progression [8]. Various studies have shown that DHIs are effective in both of these domains [67-69], although the generalizability of the findings to specific ethnic populations is limited [29]. However, studies on DHI effectiveness only describe a part of the picture. Indeed, beneficial health outcomes can only be achieved through active engagement and sustained use of the DHIs (refer to “outcomes” in Figure 2), hence the need for research to focus on the use (ie, acceptability, relevance, and user experience) of DHIs for CMDs. A recent qualitative synthesis of the available evidence has suggested that if the unmet needs (including both clinical and psychosocial or emotional) of patients with T2DM are not adequately addressed through DHIs, then the use of such DHIs will be impaired [70], which highlights the importance of patient-centered care in the digital era, a view endorsed by others [71].

Although DHIs need to ensure that they meet the digital, health, and general literacy needs of their population, some studies have challenged the assumptions about digital ownership and digital literacy in these populations [54,55]. Across the reviewed studies, 80% (12/15) of DHIs used mHealth, which reflects the growing body of research exploring the increase in mobile device adoption and use across various demographic and ethnic groups [72,73]. DHIs delivered using mobile phones were viewed positively by the participants, as they always had their mobile phones on them [54], which allowed for the integration of the interventions into their daily lives [65]. Demographic surveys in the United States, the setting of most studies included in this review, show similar levels of smartphone ownership across different ethnic groups, averaging around 83% to 85% [73]. Although comparable statistics by ethnicity do not exist for the United Kingdom, studies of internet use suggest that while >90% of people of aged ≥16 years were classified as recent internet users, there was a significant difference by age, ethnicity, and region; with Asian people aged ≥55 years were the least likely to be recent internet users [74]. However, it is important to note that individuals of White ethnicity had the highest percentage of internet nonusers [75]. In their study in resource-limited African-American communities, Yingling and colleagues [55] highlighted the similar rates of device ownership and digital literacy across study participants and point to the “new digital divide” where differences may be more pronounced among different groups, such as younger and older consumers, rather than on ethnic or socioeconomic lines. However, 47% (7/15) of the included studies provided participants with smartphones or smartphone-enabled devices [55,56,58,59,62-64], with 7% (1/15) explicitly stating this was to minimize barriers to access [63]; several studies tailored the DHIs and provided additional support to meet participants’ access needs [52-54,61,63]. This is suggestive of the ongoing digital exclusion within these low-income areas, which have not necessarily kept pace with national changes in device ownership. This further highlights the importance of engaging with the community to understand their specific needs. The coproduction of interventions with the community is likely to help guide this decision-making process.

The importance of a good design was championed across the studies included in this review. Specific features that were highlighted include incorporating pictorial and visual information [52-54,56,64,66], achieving the right balance of information volume and notifications [52,53,61,64,65], providing positive feedback for behavior change [53,54,56,59,60,64-66], progress tracking [53,54,56,60,61,64,66], cultural tailoring and representation [52-54,64,65], and the inclusion of community information and support [53,56,58,60,65,66]. Specific population needs should be considered for actions such as cultural tailoring; for example, the question of translation was only raised in the UK South Asian study [52] because English is not the first language of some participants or their networks. Despite studies showing that minority ethnic individuals have significant concerns about data sharing and surveillance in apps [76,77], only 7% (1/15) of studies in this review examined this and found that the participants were not concerned about location-based tracking [54]. Social networks were important in enabling participants’ access to DHIs [52] as well as sharing and learning with others [53], with participants in 7% (1/15) of studies wanting this to also extend offline [56]. Among South Asians, there was a great emphasis on learning from family [52,78], whereas among African-American participants, the sense of community featured more heavily, such as learning health information from their peers [29,55]. This is consistent with the finding that social support improves engagement with digital health technologies [79,80].

The role of health care professionals in delivering health education was still found to be necessary in the reviewed studies. It was suggested that although education could be incorporated into an app in the through a web-based health educator, human contact could not be entirely replaced by DHIs. This reflects the recent discussions following advances in digital technologies in health care [81]. Therefore, DHIs may offer an important adjunct rather than a replacement for usual care; the need for human support is likely to apply to other ethnic groups too, not just those included in this review [82].

Our review has several important clinical implications for the development of digital health care services aimed specifically at minority ethnic populations. Our CMOcs provide a useful guide for the future development of DHIs targeted at South Asian and Black minority ethnic populations, with specific recommendations such as user and community involvement to embed DHIs into the social context of South Asian and Black patients as well as the use of culturally competent content within DHIs. Wider considerations around the user interface and design of the DHI content to promote accessibility and inclusivity are likely to benefit broader patient demographics, regardless of their ethnic background.

### Strengths and Limitations

The strengths of our review include the use of systematic searches, the focus on CMDs for an in-depth case study of specific DHIs relevant to a high-risk population, and the inclusion of multiple researchers from different academic backgrounds, which allowed us to deepen our qualitative approach. However, we also acknowledge some important limitations of this study. First, the articles included in this review reported data collected from the United Kingdom, Canada, and the United States. Variations in the social structures of these countries, including differences in the access to and provision of health care systems, affect the generalizability of our findings across different contexts. Second, only limited evidence was found on the use of DHIs for CVD and T2DM outside the United States, with none of the identified studies focusing on the experience of people from Black, African, or Caribbean ethnic backgrounds in the United Kingdom and Canada. None of the reviewed studies included comparisons to a White control population or another minority ethnic population, which meant that the results focused on what worked in the included population and did not provide any insights into their wider applicability. Overall, the studies recruited a relatively small number of participants (n=9-343) and were limited in their ability to generalize to other socioeconomic contexts. A pragmatic approach was taken to focus on 2 minority ethnic groups for this review; further research should consider the applicability of these CMOcs to other significant minority ethnic groups in the United Kingdom and North America, for example, indigenous and Hispanic populations who are also at an increased risk of CMD [18,83], and it should also consider the heterogeneity of cultural and social factors in the broad ethnic groups considered here, as there are variations in digital use within each ethnic group [74]. In addition, other demographic factors such as socioeconomic status, education (impacting digital access and literacy), and generational differences across study participants are also likely to contribute to the use and acceptability of DHIs compared with traditional interventions [84]. Third, most of the studies we reported explored the use of mHealth, and only 7% (1/15) of studies in this review explored wearable devices; therefore, generalization to other forms of DHIs should be made with care. Although 13% (2/15) of focus group studies did not require participants to engage with DHIs as part of the study design [52,54], features related to good design were consistent across studies. Finally, there is little consistency between published realist reviews, specifically regarding the development of the IPTs and CMOcs [85]. We advocate the need to develop further guidelines for implementing realist methodologies so that future reviews are consistent and reliable.

### Conclusions

In conclusion, this review supports the use of DHIs in South Asian and Black minority ethnic populations when specific factors are considered. These include patient beliefs about the utility of DHIs and digital technology generally; education and literacy levels, the prevailing physical health needs of individual patients, social networks, material circumstances, the design of the user interface, the wider community, and the regional and national health care system. We produced CMOcs to aid future researchers in the design of cardiometabolic DHIs for minority ethnic groups.

## Appendix

Multimedia Appendix 1 Initial Programme Theories for the Implementation, Uptake, and Use of Digital Health Interventions for Cardiometabolic disease in South Asian and Black ethnic minority groups.

Multimedia Appendix 2 Inclusion and exclusion criteria.

Multimedia Appendix 3 Table showing search terms and number of results from each of the web-based databases.

## Abbreviations

CMD: cardiometabolic disease
CMOc: context-mechanism-outcome configuration
CVD: cardiovascular disease
DHI: digital health intervention
IPT: initial program theory
mHealth: mobile health
PRISMA: Preferred Reporting Items for Systematic Reviews and Meta-Analyses
RAMESES: Realist and Meta-Narrative Evidence Syntheses: Evolving Standards
T2DM: type 2 diabetes
