# Supplementary material for: The Use of Digital Health Interventions for Cardiometabolic Diseases Among South Asian and Black Minority Ethnic Groups: Realist Review
Source: J Med Internet Res. 2023 Jan 6;25:e40630. doi: 10.2196/40630 (PMC9862310; doi:10.2196/40630)
Supplement: Multimedia Appendix 1 [file jmir_v25i1e40630_app1.docx]

**Appendix 1. Initial Programme Theories for the Implementation, Uptake and Use of Digital Health Interventions for Cardiometabolic disease in South Asian and Black ethnic minority groups**

| **Individual factors i.e. factors centred on the end user** | | | |
| --- | --- | --- | --- |
| *IPT-1. Beliefs and personal views/perceptions (whether accurate or not) held around the use of the DHI itself and technology more generally will shape an individual’s uptake and use of DHIs.* | | | |
| FRAMEWORK CONSTRUCT | IMPLEMENT-  ATION (related to process) | UPTAKE (system [s] and individual [ind]) | USE (user experience, relevance, acceptability) |
| Performance expectancy (i.e. perceived usefulness)^[[1]](#footnote-2)^ |  | x^ind^ | x |
| Effort expectancy (perceived ease of use, complexity)i |  | x^ind^ | x |
| Beliefs about facilitating conditions (perceived behavioural control, facilitating conditions [organisational and technical infrastructure], compatibility)i |  | x^ind^ | x |
| Beliefs about digital health (helpful vs. harmful)^[[2]](#footnote-3)^ |  | x^ind^ | x |
| Prior use of digital resources for health seeking or health avoidanceii |  | x^ind^ | x |
| Hedonic motivation (pleasure from using technology)^[[3]](#footnote-4)^ |  | x^ind^ | x |
| Habit (previous IT use)iii |  | x^ind^ | x |
| The adopter system (patient level) (acceptability of the expectation to engage with the DHI)^[[4]](#footnote-5)^ |  | x^ind^ | x |
| Digital determinants of healthii   - Psychosocial stressors - Appraisal and coping |  | x^ind^ | x |
| *IPT-2. An individual’s education and literacy levels will affect their ability to uptake and use DHIs.* | | | |
| Digital health literacyii |  | x^ind^ | x |
| Health literacy^[[5]](#footnote-6)^ |  | x^ind^ | x |
| Computer literacyv |  | x^ind^ | x |

| *IPT-3. Prevailing physical health conditions and the associated functional disability will shape an individual’s uptake and use of DHIs.* | | | |
| --- | --- | --- | --- |
| Conditioniv   1. Comorbidities (is the patient multimorbid, functionally impaired?) 2. Sociocultural aspects of the condition (cultural stereotypes, beliefs around the condition) 3. Clinical stability of the patient 4. Typical vs atypical clinical presentation of patient 5. Cognitive reserves and health literacy of patient |  | x^ind^  x^ind^  x^ind^  x^ind^  x^ind^ | x  x  x  x  x |
| *IPT-4. An individual’s beliefs are, at least in part, determined by their social networks and the beliefs of significant others; uptake and use of DHIs is therefore shaped by social experiences/interactions.* | | | |
| Social influence (subjective norms, social factors, image)i |  | x^ind^ | x |
| The adopter system (caregiver level)iv   - Acceptability of the expectation to engage with the DHI from the point of view of the patient’s primary caregiver - Size and strength of patient’s support network to enable DHI use |  | x^ind^  x^ind^ | x  x |
| Views held by support network/caregiver regarding technology |  | x^ind^ | x |
| Social capital^[[6]](#footnote-7)^ |  | x^ind^ | x |
| *IPT-5 An individual’s material circumstances shape their uptake and use of DHIs.* | | | |
| Price value (cost and pricing structure; is there a pay wall?)iii |  | x^ind^ | x |
| Digital determinants of healthii   - Access to digital resources for health information |  | x^ind^ | x |
| Socio-economic and cultural contextsii   - Access to resources - Prestige - Discrimination |  | x^ind^  x^ind^  x^ind^ | x  x  x |
| Patient’s social position (underserved vs dominant)vi |  | x^ind^ | x |
| Material circumstancesvi |  | x^ind^ | x |
| Technology accessvi |  | x^ind^ | x |

| **Technology factors i.e. factors related to the technological aspects of the DHI** | | | |
| --- | --- | --- | --- |
| *IPT-6. The functional and performance related aspects of the DHI will shape an individual’s personal uptake and use of DHIs.* | | | |
| System features and capabilities (design characteristics of the system)^[[7]](#footnote-8)^ |  | x^ind^ | x |
| Material features (features of the DHI such as size, sounds aesthetics and dependability)iv |  | x^ind^ | x |
| Type of data generated (can the data be accepted, trusted and considered sufficient?)iv |  | x^ind^ | x |
| Knowledge needed to use (does the DHI require specialist skills to use it properly e.g. digital and health literacy)iv |  | x^ind^ | x |
| Technology supply model (is the DHI customisable or bespoke?)iv |  | x^ind^ | x |
| Elements of the DHI that enhance cultural pride^[[8]](#footnote-9)^ |  | x^ind^ | x |
| Culturally-orientated DHI contentviii |  | x^ind^ | x |
| DHI addresses people’s sociocultural and personal needsviii |  | x^ind^ | x |
| **Community factors i.e. factors related to the community in which the individual resides** | | | |
| *IPT-7. The community in which the individual lives can support the implementation, uptake and use of DHIs, if appropriately resourced.* | | | |
| Community resource systems (aspects of the community environment that can support DHI roll-out e.g. community pharmacy, social welfare assistance, parks and recreational organisations)^[[9]](#footnote-10)^ | x | x^ind^ | x |
| Co-production strategies (using individuals embedded within their community to co-create services)^[[10]](#footnote-11)^ | x | x^ind^ | x |
| Digital determinants of healthii   - Values and cultural norms/preferences for use of digital resources - Integration of digital resources into community and healthcare infrastructure | x  x | x^ind^  x^ind^ | x  x |
| Trusted sources of information (presenters from the community)viii | x | x^ind^ | x |

| **System factors i.e. factors related to the wider governing system** | | | |
| --- | --- | --- | --- |
| *IPT-8 The wider political, economic and social system of the country in which an individual resides will influence the implementation and uptake of DHIs.* | | | |
| ***Policy level system*** |  |  |  |
| Professional (professional and civil society supportive/non-supportive)iv | x | x^s^ |  |
| Political/policy context (financial and regulatory requirements nationally)iv | x | x^s^ |  |
| Regulatory/legal (medicolegal)iv | x | x^s^ |  |
| Policy (macro, social, public)vi | x | x^s^ |  |
| *IPT-9 The national and regional healthcare system in which an individual resides will influence the implementation, uptake and (in some circumstances) the use of DHIs.* | | | |
| ***Healthcare system*** |  |  |  |
| Health system as a social determinant of healthii   - Health policy - Health funding - Governance - Institutional policies and leadership - Health education and training of care providers - Patient-provider relationship | x  x  x  x  x  x | x^s^  x^s^  x^s^  x^s^  x^s, ind^  x^ind^ | x  x |
| Digital health equityii   - Involvement of people from vulnerable groups in leadership, health professions, co-design, data stewardship. - Health providers with competencies/training to provide equitable digital healthcare and necessary adaptations. - Measurement and quality improvement to improve access and outcomes. | x  x  x | x^ind^ |  |
| Resourcing and quality of careii   - Timeliness of care - Effectiveness of care - Safety of care - Person-centred care | x  x  x  x | x^ind^  x^ind^  x^s, ind^ |  |
| Health care provider systemsix   - Clinical information system (performance data related to desired outcomes) - Delivery system design (structure of teams and work groups) - Clinical decision support (integration of evidence-based guidelines into practice and reminder systems) - Patient self-management support (skill training for patients and their families). | x  x  x  x | x^s^  x^s^  x^s^  x^s, ind^ | x |
| The adopter system (staff level)   - Level of engagement by staff with the programme - Usability and ease of use of technology as viewed by the staff - Beliefs about safety and welfare of patients - Fear of job loss and scope of practice by using the technology | x  x  x  x | x^s^  x^s^  x^s^  x^s^ | x |

1. **UTAUT**: Venkatesh V, et al. User Acceptance of Information Technology: Toward a Unified View. MIS Quarterly. 2003; 27(3): 425-478 [↑](#footnote-ref-2)
2. **Digital Health Equity Framework:** Crawford A, Serhal E. Digital Health Equity and COVID-19: The Innovation Curve Cannot Reinforce the Social Gradient of Health. J Med Internet Res 2020;22(6):e19361 [↑](#footnote-ref-3)
3. **UTAUT2**: Venkatesh V, et al. Consumer Acceptance and Use of Information Technology: Extending the Unified Theory of Acceptance and Use of Technology. MIS Quarterly. 2012; 36(1):57-178 [↑](#footnote-ref-4)
4. **NASSS:** Greenhalgh T, et al. Beyond Adoption: A New Framework for Theorizing and Evaluating Nonadoption, Abandonment, and Challenges to the Scale-Up, Spread, and Sustainability of Health and Care Technologies. J Med Internet Res 2017;19(11):e367 [↑](#footnote-ref-5)
5. **Updated Integrative Model of eHealth use**: Bodie GD, et al. Understanding Health Literacy for Strategic Health Marketing: eHealth Literacy, Health Disparities, and the Digital Divide, Health Marketing Quarterly. 2008; 25(1-2):175-203 [↑](#footnote-ref-6)
6. **eHealth Equity Framework**: Antonio MG and Petrovskaya O. Towards Developing an eHealth Equity Conceptual Framework. Studies in Health Technology and Informatics. EbookVolume 257: Improving Usability, Safety and Patient Outcomes with Health Information Technology. [↑](#footnote-ref-7)
7. **TAM**: Davis, FD. Perceived usefulness, perceived ease of use and user acceptance of information technology. MIS Quarterly. 1989;13: 319-340 [↑](#footnote-ref-8)
8. **Culture-Centred TAM:** Guttman N, Lev E, Segev E, et al. “I never thought I could get health information from the Internet!”: Unexpected uses of an Internet website designed to enable Ethiopian immigrants with low/no literacy skills to browse health information. New Media & Society. 2018;20(7):2272-2295. [↑](#footnote-ref-9)
9. **CCCM**: Jenkins, Carolyn, et al. "Expanding the Chronic Care Framework to Improve Diabetes Management: The REACH Case Study." Progress in Community Health Partnerships: Research, Education, and Action, vol. 4 no. 1, 2010, p. 65-79. Project MUSE [↑](#footnote-ref-10)
10. **Matrix Framework for Co-production of Digital Services**: Kayser L, et al. Theory and Practice in Digital Behaviour Change: A Matrix Framework for the Co-Production of Digital Services That Engage, Empower and Emancipate Marginalised People Living with Complex and Chronic Conditions. Informatics. 2018; 5(4):41. [↑](#footnote-ref-11)
