# Supplementary material for: The Use of Digital Health Interventions for Cardiometabolic Diseases Among South Asian and Black Minority Ethnic Groups: Realist Review
Source: J Med Internet Res. 2023 Jan 6;25:e40630. doi: 10.2196/40630 (PMC9862310; doi:10.2196/40630)
Supplement: Multimedia Appendix 2 [file jmir_v25i1e40630_app2.docx]

**Appendix 2: Inclusion and exclusion criteria**

| Inclusion Criteria |
| --- |
| - Studies recruiting participants (a) from a South Asian or Black ethnic minority group and (b) who are at a higher risk (as defined by the study) of cardiometabolic disease, or those who have received a diagnosis of either CVD or T2DM. - DHIs relevant to cardiometabolic health (CVD or T2D or both), including eHealth and mHealth. - Qualitative or quantitative evaluations of the implementation of a specific DHI. - Studies conducted in Western, Anglophone countries. |
| Exclusion Criteria |

| - Studies examining digital health interventions unrelated to cardiometabolic health e.g. DHIs specifically developed for mental illness in the absence of a physical health condition. - Studies which explored the use of cardiometabolic DHIs designed to be used by healthcare professionals rather than the general public. - Non-English studies. - Reviews and commentary/opinion pieces with no primary data collection. - Studies from countries other than White majority, English-speaking countries. - Literature that was published over 10 years ago. - Studies that do not have a free, full-text available via UCL libraries. |
| --- |
