# Supplementary material for: The Use of Digital Health Interventions for Cardiometabolic Diseases Among South Asian and Black Minority Ethnic Groups: Realist Review
Source: J Med Internet Res. 2023 Jan 6;25:e40630. doi: 10.2196/40630 (PMC9862310; doi:10.2196/40630)
Supplement: Multimedia Appendix 3 [file jmir_v25i1e40630_app3.docx]

**Appendix 3: Table showing search terms and number of results from each of the online databases.**

| Search # | Source | Search terms | Results |
| --- | --- | --- | --- |
| 1 | Pubmed | "digital health" AND "cardio*" AND "uptake" | 10 |
| 2 | Pubmed | "digital health" AND "cardio*" AND "adoption" | 28 |
| 3 | Pubmed | "digital health" AND "cardio*" AND "use" AND "effectiveness" | 27 |
| 4 | Pubmed | "digital health" AND "cardio*" AND "use" AND "ethnicity" | 3 |
| 5 | Pubmed | "digital health" AND "cardio*" AND "ethnic*" | 8 |
| 6 | Pubmed | "digital health" AND "cardio*" AND "south asian" | 4 |
| 7 | Pubmed | "mobile" AND "cardio*" AND "south asian" | 4 |
| 8 | Pubmed | "phone" AND "cardio*" AND "south asian" | 6 |
| 9 | Pubmed | "mhealth" AND "cardio*" AND "south asian" | 1 |
| 10 | Pubmed | "digital health" AND "cardio*" AND "black" | 6 |
| 11 | Pubmed | "mhealth" AND "cardio*" AND "african" | 30 |
| 12 | Pubmed | "mhealth" AND "cardio*" AND "ethnic*" | 21 |
| 13 | Pubmed | "mhealth" AND "diabetes" AND "ethnic minorit*" | 10 |
| 14 | Pubmed | "digital" AND "diabetes" AND "ethnic minorit*" AND "use" | 6 |
| 15 | Pubmed | "telemed*" AND "diabet*" AND "south asian" | 4 |
| 16 | Pubmed | "ehealth" AND "diabet*" AND "black" | 14 |
| 17 | Pubmed | "telemed" AND "diabet*" AND "black" | 9 |
| 18 | Pubmed | "digital health" AND "diabet*" AND "ethnic" | 6 |
| 19 | Pubmed | digital health interventions uptake ethnicity | 17 |
| 20 | Pubmed | digital health interventions ethnic minorities acceptance | 25 |
| 21 | Pubmed | diabetes phone south asian | 26 |
| 22 | Pubmed | barriers diabetes management ethnic* | 165 |
| 23 | Pubmed | barriers diabetes management south asian | 32 |
| 24 | Pubmed | barriers to diabetes self management black | 86 |
| 25 | Pubmed | digital health intervention ethnicity cardiometabolic | 7 |
|  |  |  | = 555 |
| 26 | Scopus | digital  AND health  AND cardio*  AND disease  AND south  AND asian | 5 |
| 27 | Scopus | digital  AND health  AND cardio*  AND black | 27 |
| 28 | Scopus | digital  AND health  AND interventions  AND diabetes  AND adoption | 20 |
| 29 | Scopus | digital  AND health  AND use  AND cardio*  AND asian | 20 |
| 30 | Scopus | digital  AND  health  AND  adoption  AND  black | 28 |
| 31 | Scopus | "digital health"  AND  "cardiometabolic"  AND  "use"  AND  "ethnicity" | 32 |
| 32 | Scopus | "phone"  AND  "cardio*"  AND  "south asian" | 6 |
| 33 | Scopus | "phone"  AND  "cardio*"  AND  "african" | 40 |
| 34 | Scopus | diabetes  AND self  AND management  AND digital  AND health  AND ethnicity | 6 |
| 35 | Scopus | "phone"  AND  "cardio*"  AND  "black" | 25 |
| 36 | Scopus | ehealth  AND ethnicity  AND digital  AND health | 29 |
| 37 | Scopus | cardiovascular  AND ethnicity  AND digital  AND health | 21 |
| 38 | Scopus | diabetes  AND ethnicity  AND digital  AND health | 31 |
| 39 | Scopus | ethnicity  AND digital  AND health  AND interventions | 76 |
| 40 | Scopus | race  AND digital  AND health  AND interventions  AND cardio* | 4 |
| 41 | Scopus | digital  AND health  AND uptake  AND cardio* | 15 |
| 42 | Scopus | digital  AND health  AND uptake  AND diabetes | 34 |
| 43 | Scopus | digital  AND health  AND intervention  AND diabetes  AND ethnic* | 23 |
| 44 | Scopus | tech  AND health  AND diabetes  AND ethnic* | 5 |
| 45 | Scopus | digital  AND minori*  AND diabet*  AND use | 40 |
| 45 | Scopus | digital  AND health  AND uptake  AND minorit* | 13 |
| 46 | Scopus | cardiometabolic  AND digital  AND health  AND asian | 2 |
| 47 | Scopus | cardiometabolic  AND digital  AND health  AND black | 2 |
| 49 | Scopus | cardiometabolic  AND digital  AND health  AND interventions | 11 |
| 50 | Scopus | uptake AND digital  AND cardiometabolic | 2 |
|  |  |  | = 579 |
| 51 | Google Scholar | digital health interventions cvd diabetes ethnic minorities | 30 |
| 52 | Google Scholar | digital health interventions cvd diabetes african | 30 |
| 53 | Google Scholar | digital health interventions cardiometabolic | 30 |
| 54 | Google Scholar | dhis adoption ethnicity cardiovascular | 30 |
| 55 | Google Scholar | dhis usage ethnicity cardiovascular | 30 |
| 56 | Google Scholar | digital health usage minority ethnic cardiometabolic | 30 |
| 57 | Google Scholar | digital health usage minority ethnic south asian | 30 |
| 58 | Google Scholar | digital health usage minority ethnic black | 30 |
| 59 | Google Scholar | barriers to digital health ethnic minorities cvd | 30 |
| 60 | Google Scholar | barriers to digital health ethnic minorities diabetes | 30 |
| 61 | Google Scholar | barriers to digital health black cardiometabolic diseases | 30 |
| 62 | Google Scholar | adoption of dhis for cvd and diabetes in ethnic minorities | 30 |
| 63 | Google Scholar | usage digital health cvd and diabetes in south asian | 30 |
| 64 | Google Scholar | usage digital health cvd and diabetes in african | 30 |
| 65 | Google Scholar | usage digital health cvd and diabetes in ethnicity | 30 |
|  |  |  | = 450 |
